# Entanglement Purification for Logic-Qubit of Photon System Based on Parity Check Measurement Gate

**DOI:** 10.3390/e25050705

**Published:** 2023-04-24

**Authors:** Chunyan Li, Rong Kong, Baocang Ren, Meiqiu Deng, Fuguo Deng

**Affiliations:** 1 Department of Physics, National University of Defense Technology, Changsha 410073, China; 2 Hunan Key Laboratory of Mechanism and Technology of Quantum Information, Changsha 410073, China; 3 Center for Advanced Quantum Studies, Department of Physics, Beijing Normal University, Beijing 100875, China; 4 Department of Physics, Applied Optics Beijing Area Major Laboratory, Beijing Normal University, Beijing 100875, China; 5 Department of Physics, Capital Normal University, Beijing 100048, China

**Keywords:** entanglement purification, parity-check measurement gate, bit-flip error, phase-flip error

## Abstract

It has been found that logic-qubit entanglement has great potential for applications in quantum communication and quantum networks in recent years. However, along with the effects of noise and decoherence, the fidelity of the communication transmission can be greatly reduced. In this paper, we investigate the entanglement purification of logic bit-flip error and phase-flip error in polarization logic-qubit entanglement based on the parity-check measurement (PCM) gate, which is constructed by the cross-Kerr nonlinearity and used to distinguish the parity information of two-photon polarization states. The probability of entanglement purification is higher than the scheme using the linear optical method. Moreover, the quality of logic-qubit entangled states can be improved by a cyclic purification process. This entanglement purification protocol will be useful in the future when faced with long-distance communication with logic-qubit entanglement states.

## 1. Introduction

Quantum entanglement, especially maximal entanglement, plays an important role in quantum information processing (QIP), such as quantum computation [1], quantum key distribution (QKD) [2,3], quantum secure direct communication (QSDC) [4,5], quantum repeaters [6,7], quantum teleportation [8], etc. In practice, the interaction between a quantum system and environment will cause the decoherence of the quantum state, which degrades the entanglement of the quantum system or even makes it a mixed state. Therefore, the efficiency and security of quantum communication will be reduced or even fail. Several novel protocols are proposed to solve the problem and increase the communication distance, such as quantum error correction code (QECC) [9], decoherence-free subspace (DFS) [10,11,12,13,14,15], entanglement concentration [16,17,18,19,20,21,22], etc. Entanglement purification protocols (EPPs), which form the most important step in a quantum repeater, are also a powerful tool to distill higher quality entangled states from mixed states with local operation and classical communications (LOCC). The concept of entanglement purification was first proposed by Bennett et al. in 1996 [23]. In the protocol, they use the controlled-NOT (CNOT) gate, which is difficult to implement experimentally in certain photonic quantum systems at present. In 2001, Pan et al. [24] put forward an entanglement purification protocol with simple linear optical elements. Sheng et al. proposed an EPP that can be cycled to complete a higher fidelity in 2008 [25]. The deterministic EPP with finite steps using hyperentanglement state was put forward in 2010 [26,27,28]. Then, in 2014, the EPP for hyperentanglement was presented [29]. Several EPPs have been proposed in different physical systems, such as linear optical systems [30,31], spins [32,33], ionic states [34], atoms [35,36], etc.

In 2011, Fröwis and Dür described a new kind of entanglement state based on logic-qubit, named the concatenated Greenberger–Horne–Zeilinger (C-GHZ) state [37] as shown in Equation (1) [37,38,39,40,41,42,43,44,45]:

(1) $$\begin{array}{c} |\Phi^{\pm }\rangle_{N,M}=\frac{1}{\sqrt{2}}\left(|GHZ_{N}^{+}\rangle^{⊗M}\pm {|GHZ_{N}^{-}\rangle }^{⊗M}\right). \end{array}$$

where *M* is the number of logic qubits, and *N* is the number of physical qubits in each logic qubit. The logic qubit, which is a physical GHZ state, can be written as

(2) $$\begin{array}{c} |GHZ_{N}^{\pm }\rangle =\frac{1}{\sqrt{2}}\left({|H\rangle }^{⊗N}\pm {|V\rangle }^{⊗N}\right), \end{array}$$

|H〉 and |V〉 are a horizontal polarized photon and vertical polarized photon, respectively. Fröwis and Dür investigated that the C-GHZ state has its natural feature of being immune to noise [37,38,39]. In 2013, Ding et al. proposed a theoretical scheme to construct multiphoton C-GHZ states based on GHZ entanglement states [40]. In 2014, Lu et al. completed the first C-GHZ state preparation experiment in a linear optical system [41]. They also experimentally verified that the C-GHZ state can tolerate more noise than the GHZ state in a collective noisy environment. The logic Bell-state analysis of the C-GHZ state showed that the logic-qubit entanglement swapping can be performed and it could be used for long-distance quantum communication [42,43,44,45]. These theoretical and experimental studies prove that the C-GHZ state is expected to become an important quantum resource for quantum communication and large-scale quantum networks. Therefore, it is very meaningful for preserving and distilling the C-GHZ entanglement state. In 2016, Zhou and Sheng investigated the first entanglement purification protocol for a logic-qubit C-GHZ entanglement state [46]. Then, purification protocols of the C-GHZ state were proposed based on polarization [47] and linear optics [48]. In 2016, Pan et al. presented the first concentration protocol of the maximally entangled C-GHZ state with the help of polarization parity check measurement (PCM) [49]. PCM, based on the weak cross-Kerr nonlinearity [50], can be used to improve the efficiency of distilling the maximum entangled state [19,20,21,26,49,51,52,53,54,55].

In this paper, we use the PCM gate to construct the EPP of C-GHZ states with logic bit-flip error and phase-flip error. The PCM gate is constructed by the cross-Kerr nonlinearity and can be used to measure the parity information of two-photon polarization states, which allows more initial states to be purified. The quality of the mixed C-GHZ states with logic bit-flip error (or phase-flip error) can be purified by using four PCM gates, and the quality of logic-qubit entangled states can be further improved by iterating the entanglement purification process.

This paper is organized as follows. First, we briefly introduce the basic principle of the PCM with a cross-Kerr nonlinearity effect in Section 2. Then, we give a particular description of the EPPs for logic-qubits of a photon system based on PCM in Section 3. The C-GHZ state with N = 2 and M = 2 can be purified from logic bit-flip error and logic phase-flip error, respectively. In Section 4, we discuss the method of improving purification quality though iteration. At last, we give a summary in Section 5.

## 2. PCM with Weak Cross-Kerr Nonlinearity

**Figure 1:**
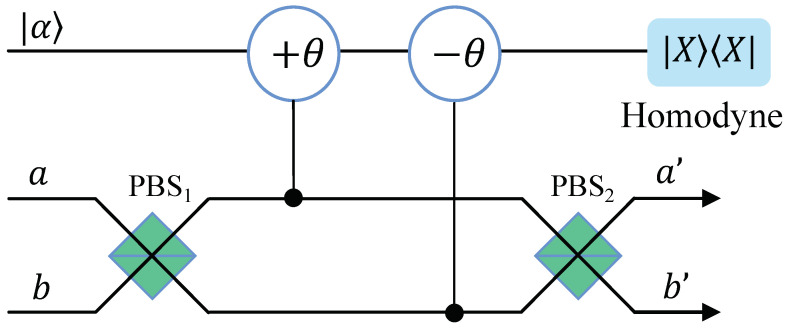
The schematic diagram of the PCM gate [43]. PBS${}_{1,2}$: polarization beam splitter; *a* and *b*: the input routes; $a^{\prime }$ and $b^{\prime }$: the output routes. Photons passing through the up (down) route will cause a phase shift −θ (+θ) on the probe coherent beam under the nonlinearity. Odd-parity and even-parity can be distinguished according to the result of homodyne measurement.

The PCM gate constructed with cross-Kerr nonlinearity has been widely used in quantum information processing because of its near determinism, such as CNOT gate [15,50], quantum clone [56], Bell state analysis [43,57], entanglement purification [26], entanglement concentration [21,49,55], etc. The cross-Kerr effect is an important mechanism in quantum technology with photonic qubits and could be achieved in a variety of quantum systems, such as trapped ions [58], artificial atoms [59], superconducting circuits [60], etc. The circuit diagram of a PCM gate is shown in Figure 1. A photon in route *a* or *b* passes through the polarization beam splitter (PBS) and acts with a probe beam in coherent state |α〉 on cross-Kerr nonlinearity. Then, it passes through another PBS to output route $a^{\prime }$ or $b^{\prime }$. The effect of PBS is to completely reflect vertically polarized photons (|V〉) and fully transmit horizontally polarized photons (|H〉). The photon collapses to a definite state according to the result of a homodyne measurement of the coherence probe beam [43]. If two photons in an arbitrarily entangled state enter the space modes *a* and *b*, respectively, the evolution of the system is shown in the following equation:

(3) $$\begin{array}{ccc} & & U_{cK}{|\varphi \rangle }_{ini}|\alpha \rangle \\ & & =e^{iH/\hbar }\left({\beta |H\rangle }_{a}{|H\rangle }_{b}+{\gamma |H\rangle }_{a}{|V\rangle }_{b}+{\delta |V\rangle }_{a}{|H\rangle }_{b}+{\varepsilon |V\rangle }_{a}{|V\rangle }_{b}\right)|\alpha \rangle \\ & & =\left({\beta |H\rangle }_{a^{\prime }}{|H\rangle }_{b^{\prime }}+{\varepsilon |V\rangle }_{a^{\prime }}{|V\rangle }_{b^{\prime }}\right)|\alpha \rangle +{\gamma |H\rangle }_{a^{\prime }}{|V\rangle }_{b^{\prime }}|\alpha e^{-i2\theta }{\rangle +\delta |V\rangle }_{a^{\prime }}{|H\rangle }_{b^{\prime }}|\alpha e^{i2\theta }\rangle . \end{array}$$

where ${|\varphi \rangle }_{ini}$ is the initial state of two signal photons. The coefficients β, γ, δ, and ε satisfy the normalization condition ${\left|\beta \right|}^{2}+{\left|\gamma \right|}^{2}+{\left|\delta \right|}^{2}+{\left|\varepsilon \right|}^{2}=1$. $H=\hbar \chi {\hat{n}}_{s}{\hat{n}}_{p}$ is the evolution Hamiltonian of the cross-Kerr nonlinear interaction between a single particle and the probe coherent beam. ${\hat{n}}_{s(p)}$ is the photon number operator of the single (probe) beam, and χ is the nonlinearity strength, which is determined by the nonlinear material. θ=χt is the phase shift of the coherent beam caused by the signal photon, where *t* is the interaction time [50].

As shown in Equation (3), the state of the signal of the photon will collapse to an even parity state ${\beta |H\rangle }_{a^{\prime }}{|H\rangle }_{b^{\prime }}+{\varepsilon |V\rangle }_{a^{\prime }}{|V\rangle }_{b^{\prime }}$ superimposed by |HH〉 and |VV〉 when there is no phase shift in the coherent state. In order to make no distinction between states with tiny phase shifts +2θ and −2θ, we can use X quadrature homodyne measurement instead of a homodyne–heterodyne measurement, as shown in [50,57]. Then, the state will collapse to an odd parity state ${\gamma |H\rangle }_{a^{\prime }}{|V\rangle }_{b^{\prime }}+{\delta |V\rangle }_{a^{\prime }}{|H\rangle }_{b^{\prime }}$ superimposed by |HV〉 and |VH〉 when there is a phase shift of ±2θ in the coherent state. The error probability of distinguishing the phase shift 0 and ±2θ is $P_{error}=\frac{1}{2}erfc\left(\alpha \theta^{2}/2\sqrt{2}\right)$, which is less than $10^{-5}$ when $\alpha \theta^{2}>9$ [50].

## 3. Entanglement Purification for Logic-Qubit of Photon System Based on PCM Gate

If two parties, Alice and Bob, want to purify the logic C-GHZ state, $|\Phi^{+}\rangle_{AB}$ with N=M=2 in Equation (1). The logic entanglement state in this case is described as

(4) $$\begin{array}{c} |\Phi^{+}\rangle_{AB}=\frac{1}{\sqrt{2}}\left(|\phi^{+}\rangle_{A}|\phi^{+}\rangle_{B}+|\phi^{-}\rangle_{A}{|\phi^{-}\rangle }_{B}\right)\;. \end{array}$$

Here, the subscripts A and B distinguish photons belonging to Alice and Bob, respectively. $|\phi^{\pm }\rangle_{AB}$ are two maximally entangled states of the four polarization Bell states, which can be written as

(5) $$\begin{array}{c} |\phi^{\pm }\rangle_{1,2}=\frac{1}{\sqrt{2}}\left({|H\rangle }_{1}{|H\rangle }_{2}\pm {|V\rangle }_{1}{|V\rangle }_{2}\right)\;,\\ |\psi^{\pm }\rangle_{1,2}=\frac{1}{\sqrt{2}}\left({|H\rangle }_{1}{|V\rangle }_{2}\pm {|V\rangle }_{1}{|H\rangle }_{2}\right)\;. \end{array}$$

The subscripts 1 and 2 note the spatial modes of photons.

### 3.1. The Purification of the Logic Bit-Flip Error

If the photons make a logic bit-flip error due to the channel noise with a probability of 1−F, the state $|\Phi^{+}\rangle_{AB}$ can be changed into the following state as

(6) $$\begin{array}{c} |\Psi^{+}\rangle_{AB}=\frac{1}{\sqrt{2}}\left(|\phi^{+}\rangle_{A}|\phi^{-}\rangle_{B}+|\phi^{-}\rangle_{A}{|\phi^{+}\rangle }_{B}\right)\;. \end{array}$$

Alice and Bob will obtain the following mixed entanglement states:

(7) $$\begin{array}{c} \rho_{0}=F|\Phi^{+}\rangle \langle \Phi^{+}\left|+\left(1-F\right)\right|\Psi^{+}\rangle \langle \Psi^{+}|. \end{array}$$

**Figure 2:**
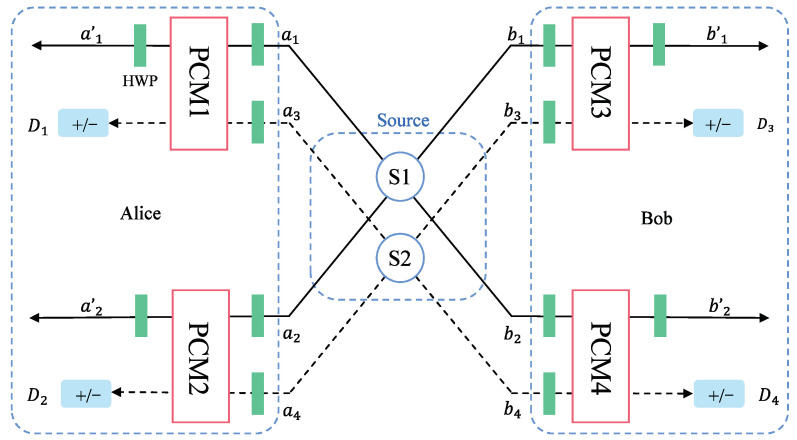
Schematic diagram of the purification of the logic fit-flip error based on PCM gates. *S*: entanglement source; HWP: half wave plate; PCM: parity check measurement gate; *a* and *b*: the input routes; $a^{\prime }$ and $b^{\prime }$: the output routes. *D*: single photon detector that can distinguish between states |+〉 and |−〉.

The schematic diagram of the entanglement purification of the logic bit-flip error based on the PCM gate is shown in Figure 2. In order to purify the maximum entangled state $|\Phi^{+}\rangle_{AB}$ from Equation (7), Alice and Bob first share two copies of initial mixed states $\rho_{1}$ and $\rho_{2}$ from the source S. The whole system can be described as $\rho_{1}⊗\rho_{2}$. Photons are in states $|\Phi^{+}\rangle_{A1B1}⊗{|\Phi^{+}\rangle }_{A2B2}$, $|\Phi^{+}\rangle_{A1B1}⊗{|\Psi^{+}\rangle }_{A2B2}$, $|\Psi^{+}\rangle_{A1B1}⊗{|\Phi^{+}\rangle }_{A2B2}$ and $|\Psi^{+}\rangle_{A1B1}⊗{|\Psi^{+}\rangle }_{A2B2}$ with probabilities $F^{2}$, F(1−F), F(1−F) and ${\left(1-F\right)}^{2}$, respectively. The photons of state $\rho_{1}$ and $\rho_{2}$ are in spatial mode $\left\{a_{1},a_{2},b_{1},b_{2}\right\}$ and $\left\{a_{3},a_{4},b_{3},b_{4}\right\}$, respectively. As shown in Figure 2, $\left\{a_{1},a_{2},a_{3},a_{4}\right\}$ belongs to Alice and $\left\{b_{1},b_{2},b_{3},b_{4}\right\}$ belongs to Bob.

Alice and Bob first make each photon pass through a half-wave plate (HWP), which makes $|H\rangle \to \frac{1}{\sqrt{2}}\left(|H\rangle +|V\rangle \right)$ and $|V\rangle \to \frac{1}{\sqrt{2}}\left(|H\rangle -|V\rangle \right)$. The eight HWPs will transform the states in Equation (5) as follows:

(8) $$\begin{array}{c} |\phi^{+}\rangle \to |\phi^{+}\rangle ;\;|\phi^{-}\rangle \to |\psi^{+}\rangle ;\;|\psi^{+}\rangle \to |\phi^{-}\rangle ;\;|\psi^{-}\rangle \to |\psi^{-}\rangle . \end{array}$$

In the following, we take $|\Phi^{+}\rangle_{A1B1}⊗{|\Phi^{+}\rangle }_{A2B2}$ as an example to discuss the evolution of quantum states in detail. The state of photons passing through the HWPs can be described as follows.

$$\begin{array}{ccc} \; & & \frac{1}{\sqrt{2}}\left(|\phi^{+}\rangle_{A1}|\phi^{+}\rangle_{B1}+|\psi^{+}\rangle_{A1}{|\psi^{+}\rangle }_{B1}\right)⊗\frac{1}{\sqrt{2}}\left(|\phi^{+}\rangle_{A2}|\phi^{+}\rangle_{B2}+|\psi^{+}\rangle_{A2}{|\psi^{+}\rangle }_{B2}\right)\\ & = & \frac{1}{\sqrt{2}}[\frac{1}{\sqrt{2}}\left({|H\rangle }_{a_{1}}{|H\rangle }_{a_{2}}+{|V\rangle }_{a_{1}}{|V\rangle }_{a_{2}}\right)⊗\frac{1}{\sqrt{2}}\left({|H\rangle }_{b_{1}}{|H\rangle }_{b_{2}}+{|V\rangle }_{b_{1}}{|V\rangle }_{b_{2}}\right)\\ & & +\frac{1}{\sqrt{2}}\left({|H\rangle }_{a_{1}}{|V\rangle }_{a_{2}}+{|V\rangle }_{a_{1}}{|H\rangle }_{a_{2}}\right)⊗\frac{1}{\sqrt{2}}\left({|H\rangle }_{b_{1}}{|V\rangle }_{b_{2}}+{|V\rangle }_{b_{1}}{|H\rangle }_{b_{2}}\right)]\\ & & ⊗\frac{1}{\sqrt{2}}[\frac{1}{\sqrt{2}}\left({|H\rangle }_{a_{3}}{|H\rangle }_{a_{4}}+{|V\rangle }_{a_{3}}{|V\rangle }_{a_{4}}\right)⊗\frac{1}{\sqrt{2}}\left({|H\rangle }_{b_{3}}{|H\rangle }_{b_{4}}+{|V\rangle }_{b_{3}}{|V\rangle }_{b_{4}}\right)\\ & & +\frac{1}{\sqrt{2}}\left({|H\rangle }_{a_{3}}{|V\rangle }_{a_{4}}+{|V\rangle }_{a_{3}}{|H\rangle }_{a_{4}}\right)⊗\frac{1}{\sqrt{2}}\left({|H\rangle }_{b_{3}}{|V\rangle }_{b_{4}}+{|V\rangle }_{b_{3}}{|H\rangle }_{b_{4}}\right)] \end{array}$$

Eight photons enter the PCM gates in four groups, $\left\{a_{1},a_{3}\right\}$, $\left\{a_{2},a_{4}\right\}$, $\left\{b_{1},b_{3}\right\}$ and $\left\{b_{2},b_{4}\right\}$. The PCM results of all initial states are shown in Table 1. Numbers 0 and 1 in the table mark the PCM results of even-parity and odd-parity, respectively. From the table, we can easily distinguish $|\Phi^{+}\rangle_{A1B1}⊗{|\Phi^{+}\rangle }_{A2B2}$ and $|\Psi^{+}\rangle_{A1B1}⊗{|\Psi^{+}\rangle }_{A2B2}$ from $|\Phi^{+}\rangle_{A1B1}⊗{|\Psi^{+}\rangle }_{A2B2}$ and $|\Psi^{+}\rangle_{A1B1}⊗{|\Phi^{+}\rangle }_{A2B2}$ according to the PCMs. If the four PCM gates have an even number of even-parity or odd-parity results, such as {0000,1111,0011,1100,1001,0110,1010,0101}, the following purification process is continued.

For example, if the result of PCM is 0000 with the initial state $|\Phi^{+}\rangle_{A1B1}⊗{|\Phi^{+}\rangle }_{A2B2}$, the unnormalized state of the photons is as follows:

(9) $$\begin{array}{ccc} & & \frac{1}{8}[\left({|H\rangle }_{a_{1}^{\prime }}{|H\rangle }_{a_{3}^{\prime }}{|H\rangle }_{a_{2}^{\prime }}{|H\rangle }_{a_{4}^{\prime }}+{|V\rangle }_{a_{1}^{\prime }}{|V\rangle }_{a_{3}^{\prime }}{|V\rangle }_{a_{2}^{\prime }}{|V\rangle }_{a_{4}^{\prime }}\right)\\ & & ⊗\left({|H\rangle }_{b_{1}^{\prime }}{|H\rangle }_{b_{3}^{\prime }}{|H\rangle }_{b_{2}^{\prime }}{|H\rangle }_{b_{4}^{\prime }}+{|V\rangle }_{b_{1}^{\prime }}{|V\rangle }_{b_{3}^{\prime }}{|V\rangle }_{b_{2}^{\prime }}{|V\rangle }_{b_{4}^{\prime }}\right)\\ & + & \left({|H\rangle }_{a_{1}^{\prime }}{|H\rangle }_{a_{3}^{\prime }}{|V\rangle }_{a_{2}^{\prime }}{|V\rangle }_{a_{4}^{\prime }}+{|V\rangle }_{a_{1}^{\prime }}{|V\rangle }_{a_{3}^{\prime }}{|H\rangle }_{a_{2}^{\prime }}{|H\rangle }_{a_{4}^{\prime }}\right)\\ & & ⊗\left({|H\rangle }_{b_{1}^{\prime }}{|H\rangle }_{b_{3}^{\prime }}{|V\rangle }_{b_{2}^{\prime }}{|V\rangle }_{b_{4}^{\prime }}+{|V\rangle }_{b_{1}^{\prime }}{|V\rangle }_{b_{3}^{\prime }}{|H\rangle }_{b_{2}^{\prime }}{|H\rangle }_{b_{4}^{\prime }}\right)]. \end{array}$$

If the result of PCM is 0000 with the initial state $|\Psi^{+}\rangle_{A1B1}⊗{|\Psi^{+}\rangle }_{A2B2}$, the unnormalized state of the photons is

(10) $$\begin{array}{ccc} & & \frac{1}{8}[\left({|H\rangle }_{a_{1}^{\prime }}{|H\rangle }_{a_{3}^{\prime }}{|H\rangle }_{a_{2}^{\prime }}{|H\rangle }_{a_{4}^{\prime }}+{|V\rangle }_{a_{1}^{\prime }}{|V\rangle }_{a_{3}^{\prime }}{|V\rangle }_{a_{2}^{\prime }}{|V\rangle }_{a_{4}^{\prime }}\right)\\ & & ⊗\left({|H\rangle }_{b_{1}^{\prime }}{|H\rangle }_{b_{3}^{\prime }}{|V\rangle }_{b_{2}^{\prime }}{|V\rangle }_{b_{4}^{\prime }}+{|V\rangle }_{b_{1}^{\prime }}{|V\rangle }_{b_{3}^{\prime }}{|H\rangle }_{b_{2}^{\prime }}{|H\rangle }_{b_{4}^{\prime }}\right)\\ & + & \left({|H\rangle }_{a_{1}^{\prime }}{|H\rangle }_{a_{3}^{\prime }}{|V\rangle }_{a_{2}^{\prime }}{|V\rangle }_{a_{4}^{\prime }}+{|V\rangle }_{a_{1}^{\prime }}{|V\rangle }_{a_{3}^{\prime }}{|H\rangle }_{a_{2}^{\prime }}{|H\rangle }_{a_{4}^{\prime }}\right)\\ & & ⊗\left({|H\rangle }_{b_{1}^{\prime }}{|H\rangle }_{b_{3}^{\prime }}{|H\rangle }_{b_{2}^{\prime }}{|H\rangle }_{b_{4}^{\prime }}+{|V\rangle }_{b_{1}^{\prime }}{|V\rangle }_{b_{3}^{\prime }}{|V\rangle }_{b_{2}^{\prime }}{|V\rangle }_{b_{4}^{\prime }}\right)]. \end{array}$$

In order to obtain the probability of success more clearly, the coefficients in Equations (10) and (11) are not normalized. Then, Alice and Bob let photons in spatial modes $\left\{a_{1}^{\prime },a_{2}^{\prime },b_{1}^{\prime },b_{2}^{\prime }\right\}$ pass through HWPs and make measurements in “+/−” bases to photons in spatial modes $\left\{a_{3}^{\prime },a_{4}^{\prime },b_{3}^{\prime },b_{4}^{\prime }\right\}$ by special single photon detectors (SPDs). “+” and “−” bases correspond to the collapse of photons into states $|+\rangle =\frac{1}{\sqrt{2}}(|H\rangle +\left|V\rangle \right)$ and $|+\rangle =\frac{1}{\sqrt{2}}(|H\rangle -\left|V\rangle \right)$. If the measurement results of detectors in spatial modes $\left\{a_{3}^{\prime },a_{4}^{\prime },b_{3}^{\prime },b_{4}^{\prime }\right\}$ are “++++” or “−−−−”, the initial quantum states $|\Phi^{+}\rangle_{A1B1}⊗{|\Phi^{+}\rangle }_{A2B2}$ and $|\Psi^{+}\rangle_{A1B1}⊗{|\Psi^{+}\rangle }_{A2B2}$ collapse to states $|\Phi^{\prime +}\rangle_{AB}$ and $|\Psi^{\prime +}\rangle_{AB}$, as shown in Equations (11) and (12) after HWPs, which means the entanglement purification is successful.

(11) $$\begin{array}{c} |\Phi^{\prime +}\rangle_{AB}=\frac{1}{\sqrt{2}}\left(|\phi^{+}\rangle_{a_{1}^{\prime }a_{2}^{\prime }}|\phi^{+}\rangle_{b_{1}^{\prime }b_{2}^{\prime }}+|\phi^{-}\rangle_{a_{1}^{\prime }a_{2}^{\prime }}{|\phi^{-}\rangle }_{b_{1}^{\prime }b_{2}^{\prime }}\right), \end{array}$$

(12) $$\begin{array}{c} |\Psi^{\prime +}\rangle_{AB}=\frac{1}{\sqrt{2}}\left(|\phi^{+}\rangle_{a_{1}^{\prime }a_{2}^{\prime }}|\phi^{-}\rangle_{b_{1}^{\prime }b_{2}^{\prime }}+|\phi^{-}\rangle_{a_{1}^{\prime }a_{2}^{\prime }}{|\phi^{+}\rangle }_{b_{1}^{\prime }b_{2}^{\prime }}\right). \end{array}$$

According to the above analysis, 1/64 of the initial states was successfully purified based on PCM and the ideal entanglement source. With regard to the other measurement results of SPDs, the two users can perform proper operations to improve the probability of entanglement purification, as shown in Table 2. The probability of the users getting state $|\Phi^{\prime +}\rangle$ and state $|\Psi^{\prime +}\rangle$ is increased to $F^{2}/8$ and ${\left(1-F\right)}^{2}/8$, respectively. The other seven PCMs results with initial states $|\Phi^{+}\rangle_{A1B1}⊗{|\Phi^{+}\rangle }_{A2B2}$ and $|\Psi^{+}\rangle_{A1B1}⊗{|\Psi^{+}\rangle }_{A2B2}$ in Table 1 have the same operations to restore to the input state as shown in Table 2. Therefore, the probability that users share entangled state $|\Phi^{\prime +}\rangle$ and $|\Psi^{\prime +}\rangle$ after purification is $F^{2}$ and ${\left(1-F\right)}^{2}$, respectively. The probabilities are $F^{2}/8$ and ${\left(1-F\right)}^{2}/8$ in the purification scheme using linear optical methods [48]. Because both of the two kinds of initial entangled states can be purified, our scheme has a higher success probability.

After this entanglement purification process, the two entanglement photon systems have no bit-flip errors, and the events that both result in bit-flip errors are preserved in ideal conditions. The output mixed state can be written as

(13) $$\begin{array}{c} \rho_{out}^{\prime }=F^{\prime }|\Phi^{\prime +}\rangle \langle \Phi^{\prime +}|+\left(1-F^{\prime }\right)|\Psi^{\prime +}\rangle \langle \Psi^{\prime +}|. \end{array}$$

The new fidelity of the photon pairs without bit-flip errors becomes

(14) $$\begin{array}{c} F^{\prime }=\frac{F^{2}}{F^{2}+\left(1-F^{2}\right)}, \end{array}$$

The purification is successful when F>1.

### 3.2. The Purification of the Logic Phase-Flip Error

The state $|\Phi^{+}\rangle_{AB}$ can be changed into the following state when the photons make a logic phase-flip error with the possibility of 1−F.

(15) $$\begin{array}{c} |\Phi^{-}\rangle_{AB}=\frac{1}{\sqrt{2}}\left(|\phi^{+}\rangle_{A}|\phi^{+}\rangle_{B}-|\phi^{-}\rangle_{A}{|\phi^{-}\rangle }_{B}\right)\;. \end{array}$$

The density matrix of the mixed entanglement states is as follows:

(16) $$\begin{array}{c} \rho_{0}=F|\Phi^{+}\rangle \langle \Phi^{+}\left|+\left(1-F\right)\right|\Phi^{-}\rangle \langle \Phi^{-}|. \end{array}$$

**Figure 3:**
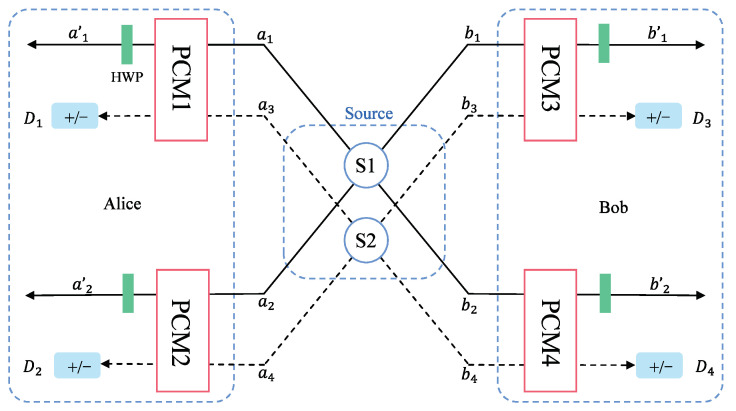
Schematic diagram of the purification of the logic phase-flip error based on PCM gates. *S*: entanglement source; HWP: half-wave plate; PCM: parity check measurement gate; *a* and *b*: the input routes; $a^{\prime }$ and $b^{\prime }$: the output routes. *D*: single photon detector that can distinguish between states |+〉 and |−〉.

The schematic diagram of the entanglement purification of the logic phase-flip error is shown in Figure 3. The difference with the purified bit-flip error is that photons do not need to pass through the HWPs before entering the PCMs. Before purification, Alice and Bob share two copies of mixed states in Equation (16) from source S, denoted as $\rho_{1}⊗\rho_{2}$. It is in the state $|\Phi^{+}\rangle_{A1B1}⊗{|\Phi^{+}\rangle }_{A2B2}$, $|\Phi^{+}\rangle_{A1B1}⊗{|\Phi^{-}\rangle }_{A2B2}$, $|\Phi^{-}\rangle_{A1B1}⊗{|\Phi^{+}\rangle }_{A2B2}$, and $|\Phi^{-}\rangle_{A1B1}⊗{|\Phi^{-}\rangle }_{A2B2}$ with probability $F^{2}$, F(1−F), F(1−F), and ${\left(1-F\right)}^{2}$, respectively. The photons of state $\rho_{1}$ and $\rho_{2}$ are in spatial modes $\left\{a_{1},a_{2},b_{1},b_{2}\right\}$ and $\left\{a_{3},a_{4},b_{3},b_{4}\right\}$, respectively.

As shown in Figure 3, photons sent to Alice in spatial modes $\left\{a_{1},a_{3}\right\}$ and $\left\{a_{2},a_{4}\right\}$ pass through PCM1 and PCM2 gates, respectively, and photons belonging to Bob in spatial modes $\left\{b_{1},b_{3}\right\}$ and $\left\{b_{2},b_{4}\right\}$ pass through PCM3 and PCM4 gates, respectively. According to the results of polarization parity check measurement, $|\Phi^{+}\rangle_{A1B1}⊗{|\Phi^{+}\rangle }_{A2B2}$ and $|\Phi^{-}\rangle_{A1B1}⊗{|\Phi^{-}\rangle }_{A2B2}$ can be distinguished from $|\Phi^{+}\rangle_{A1B1}⊗{|\Phi^{-}\rangle }_{A2B2}$ and $|\Phi^{-}\rangle_{A1B1}⊗{|\Phi^{+}\rangle }_{A2B2}$ (see in Table 3).

If the result of PCM{1,2,3,4} is 0000 or 1111, the state of photons will collapse to the state as shown below.

(17) $$\begin{array}{ccc} & & 0000:\\ & & \;|\Phi^{+}\rangle_{A1B1}⊗{|\Phi^{+}\rangle }_{A2B2}\to \\ & & \;\;\frac{1}{4}{[{(|H\rangle }_{a_{1}^{\prime }}|H\rangle }_{a_{2}^{\prime }}{|H\rangle }_{a_{3}^{\prime }}{|H\rangle }_{a_{4}^{\prime }}+{|V\rangle }_{a_{1}^{\prime }}{|V\rangle }_{a_{2}^{\prime }}{|V\rangle }_{a_{3}^{\prime }}{|V\rangle }_{a_{4}^{\prime }})\\ & & ⊗{(|H\rangle }_{b_{1}^{\prime }}{|H\rangle }_{b_{2}^{\prime }}{|H\rangle }_{b_{3}^{\prime }}{|H\rangle }_{b_{4}^{\prime }}+{|V\rangle }_{b_{1}^{\prime }}{|V\rangle }_{b_{2}^{\prime }}{|V\rangle }_{b_{3}^{\prime }}{|V\rangle }_{b_{4}^{\prime }})\\ & & \;+{(|H\rangle }_{a_{1}^{\prime }}{|H\rangle }_{a_{2}^{\prime }}{|H\rangle }_{a_{3}^{\prime }}{|H\rangle }_{a_{4}^{\prime }}-{|V\rangle }_{a_{1}^{\prime }}{|V\rangle }_{a_{2}^{\prime }}{|V\rangle }_{a_{3}^{\prime }}{|V\rangle }_{a_{4}^{\prime }})\\ & & ⊗{(|H\rangle }_{b_{1}^{\prime }}{|H\rangle }_{b_{2}^{\prime }}{|H\rangle }_{b_{3}^{\prime }}{|H\rangle }_{b_{4}^{\prime }}-{|V\rangle }_{b_{1}^{\prime }}{|V\rangle }_{b_{2}^{\prime }}{|V\rangle }_{b_{3}^{\prime }}{|V\rangle }_{b_{4}^{\prime }})], \end{array}$$

(18) $$\begin{array}{ccc} & & \;|\Phi^{-}\rangle_{A1B1}⊗{|\Phi^{-}\rangle }_{A2B2}\to \\ & & \;\;\frac{1}{4}{[{(|H\rangle }_{a_{1}^{\prime }}|H\rangle }_{a_{2}^{\prime }}{|H\rangle }_{a_{3}^{\prime }}{|H\rangle }_{a_{4}^{\prime }}+{|V\rangle }_{a_{1}^{\prime }}{|V\rangle }_{a_{2}^{\prime }}{|V\rangle }_{a_{3}^{\prime }}{|V\rangle }_{a_{4}^{\prime }})\\ & & ⊗{(|H\rangle }_{b_{1}^{\prime }}{|H\rangle }_{b_{2}^{\prime }}{|H\rangle }_{b_{3}^{\prime }}{|H\rangle }_{b_{4}^{\prime }}+{|V\rangle }_{b_{1}^{\prime }}{|V\rangle }_{b_{2}^{\prime }}{|V\rangle }_{b_{3}^{\prime }}{|V\rangle }_{b_{4}^{\prime }})\\ & & \;-{(|H\rangle }_{a_{1}^{\prime }}{|H\rangle }_{a_{2}^{\prime }}{|H\rangle }_{a_{3}^{\prime }}{|H\rangle }_{a_{4}^{\prime }}-{|V\rangle }_{a_{1}^{\prime }}{|V\rangle }_{a_{2}^{\prime }}{|V\rangle }_{a_{3}^{\prime }}{|V\rangle }_{a_{4}^{\prime }})\\ & & ⊗{(|H\rangle }_{b_{1}^{\prime }}{|H\rangle }_{b_{2}^{\prime }}{|H\rangle }_{b_{3}^{\prime }}{|H\rangle }_{b_{4}^{\prime }}-{|V\rangle }_{b_{1}^{\prime }}{|V\rangle }_{b_{2}^{\prime }}{|V\rangle }_{b_{3}^{\prime }}{|V\rangle }_{b_{4}^{\prime }})], \end{array}$$

(19) $$\begin{array}{ccc} & & 1111:\\ & & \;|\Phi^{+}\rangle_{A1B1}⊗{|\Phi^{+}\rangle }_{A2B2}\to \\ & & \frac{1}{4}{[{(|H\rangle }_{a_{1}^{\prime }}|H\rangle }_{a_{2}^{\prime }}{|V\rangle }_{a_{3}^{\prime }}{|V\rangle }_{a_{4}^{\prime }}+{|V\rangle }_{a_{1}^{\prime }}{|V\rangle }_{a_{2}^{\prime }}{|H\rangle }_{a_{3}^{\prime }}{|H\rangle }_{a_{4}^{\prime }})\\ & & ⊗{(|H\rangle }_{b_{1}^{\prime }}{|H\rangle }_{b_{2}^{\prime }}{|V\rangle }_{b_{3}^{\prime }}{|V\rangle }_{b_{4}^{\prime }}+{|V\rangle }_{b_{1}^{\prime }}{|V\rangle }_{b_{2}^{\prime }}{|H\rangle }_{b_{3}^{\prime }}{|H\rangle }_{b_{4}^{\prime }})\\ & & +{(|H\rangle }_{a_{1}^{\prime }}{|H\rangle }_{a_{2}^{\prime }}{|V\rangle }_{a_{3}^{\prime }}{|V\rangle }_{a_{4}^{\prime }}-{|V\rangle }_{a_{1}^{\prime }}{|V\rangle }_{a_{2}^{\prime }}{|H\rangle }_{a_{3}^{\prime }}{|H\rangle }_{a_{4}^{\prime }})\\ & & ⊗{(|H\rangle }_{b_{1}^{\prime }}{|H\rangle }_{b_{2}^{\prime }}{|V\rangle }_{b_{3}^{\prime }}{|V\rangle }_{b_{4}^{\prime }}-{|V\rangle }_{b_{1}^{\prime }}{|V\rangle }_{b_{2}^{\prime }}{|H\rangle }_{b_{3}^{\prime }}{|H\rangle }_{b_{4}^{\prime }})]. \end{array}$$

(20) $$\begin{array}{ccc} & & \;|\Phi^{-}\rangle_{A1B1}⊗{|\Phi^{-}\rangle }_{A2B2}\to \\ & & \frac{1}{4}{[{(|H\rangle }_{a_{1}^{\prime }}|H\rangle }_{a_{2}^{\prime }}{|V\rangle }_{a_{3}^{\prime }}{|V\rangle }_{a_{4}^{\prime }}+{|V\rangle }_{a_{1}^{\prime }}{|V\rangle }_{a_{2}^{\prime }}{|H\rangle }_{a_{3}^{\prime }}{|H\rangle }_{a_{4}^{\prime }})\\ & & ⊗{(|H\rangle }_{b_{1}^{\prime }}{|H\rangle }_{b_{2}^{\prime }}{|V\rangle }_{b_{3}^{\prime }}{|V\rangle }_{b_{4}^{\prime }}+{|V\rangle }_{b_{1}^{\prime }}{|V\rangle }_{b_{2}^{\prime }}{|H\rangle }_{b_{3}^{\prime }}{|H\rangle }_{b_{4}^{\prime }})\\ & & -{(|H\rangle }_{a_{1}^{\prime }}{|H\rangle }_{a_{2}^{\prime }}{|V\rangle }_{a_{3}^{\prime }}{|V\rangle }_{a_{4}^{\prime }}-{|V\rangle }_{a_{1}^{\prime }}{|V\rangle }_{a_{2}^{\prime }}{|H\rangle }_{a_{3}^{\prime }}{|H\rangle }_{a_{4}^{\prime }})\\ & & ⊗{(|H\rangle }_{b_{1}^{\prime }}{|H\rangle }_{b_{2}^{\prime }}{|V\rangle }_{b_{3}^{\prime }}{|V\rangle }_{b_{4}^{\prime }}-{|V\rangle }_{b_{1}^{\prime }}{|V\rangle }_{b_{2}^{\prime }}{|H\rangle }_{b_{3}^{\prime }}{|H\rangle }_{b_{4}^{\prime }})]. \end{array}$$

The coefficients in Equations (18)–(21) are not normalized. The following process is the same as the entanglement purification process of the logic bit-error. The entanglement purification is successful when the measurement results of detectors $\left\{D_{1},D_{2},D_{3},D_{4}\right\}$ have even “+” or “−”, as shown in Table 4. The state of the photons in spatial modes $\left\{a_{1}^{\prime },a_{2}^{\prime },b_{1}^{\prime },b_{2}^{\prime }\right\}$ will collapse to state $|\Phi^{\prime +}\rangle_{AB}$ or $|\Phi^{\prime -}\rangle_{AB}$ as Equations (22) and (23) according to the initial state.

(21) $$\begin{array}{c} |\Phi^{\prime +}\rangle_{AB}=\frac{1}{\sqrt{2}}\left(|\phi^{+}\rangle_{a_{1}^{\prime }a_{2}^{\prime }}|\phi^{+}\rangle_{b_{1}^{\prime }b_{2}^{\prime }}+|\phi^{-}\rangle_{a_{1}^{\prime }a_{2}^{\prime }}{|\phi^{-}\rangle }_{b_{1}^{\prime }b_{2}^{\prime }}\right), \end{array}$$

(22) $$\begin{array}{c} |\Phi^{\prime -}\rangle_{AB}=\frac{1}{\sqrt{2}}\left(|\phi^{+}\rangle_{a_{1}^{\prime }a_{2}^{\prime }}|\phi^{+}\rangle_{b_{1}^{\prime }b_{2}^{\prime }}-|\phi^{-}\rangle_{a_{1}^{\prime }a_{2}^{\prime }}{|\phi^{-}\rangle }_{b_{1}^{\prime }b_{2}^{\prime }}\right). \end{array}$$

When the measurement result of $D_{1},D_{2},D_{3},D_{3}$ is one of the odd “+” or “−” situations, as shown in Table 4, the state can be restored to the input state through the unitary operation “$\sigma_{z}$” on the photon of path $b_{1}^{\prime }$ by Bob. Finally, the probabilities that Alice and Bob share states $|\Phi^{\prime +}\rangle$ and $|\Phi^{\prime -}\rangle$ are $F^{2}$ and ${\left(1-F\right)}^{2}$, respectively. The output mixed state after entanglement purification is

(23) $$\begin{array}{c} \rho_{out}^{\prime }=F^{\prime }|\Phi^{\prime +}\rangle \langle \Phi^{\prime +}|+\left(1-F^{\prime }\right)|\Phi^{\prime -}\rangle \langle \Phi^{\prime -}|, \end{array}$$

where $F^{\prime }=\frac{F^{2}}{F^{2}+{\left(1-F\right)}^{2}}$ is the new fidelity of the state without bit-flip errors. When F>1, the quality of the logic-qubit entangled state is improved, and the phase-flip error is purified.

## 4. Improving the Fidelity by Circulation Purification

We have discussed the purification process for four photons’ C-GHZ entanglement states with logic bit-flip error and phase-flip error, respectively. After purification, both kinds of errors have the same fidelity $\frac{F^{2}}{F^{2}+\left(1-F^{2}\right)}$, which is larger than the fidelity of the initial state under the situation $F>\frac{1}{2}$. It is easy to see that the purified mixed state can be used as the initial state for further purification to obtain greater fidelity. The fidelity in theory after *n* cycles of purification is

(24) $$\begin{array}{c} F^{\left(n\right)}=\frac{F^{2^{n}}}{F^{2^{n}}+{\left(1-F\right)}^{2^{n}}}. \end{array}$$

**Figure 4:**
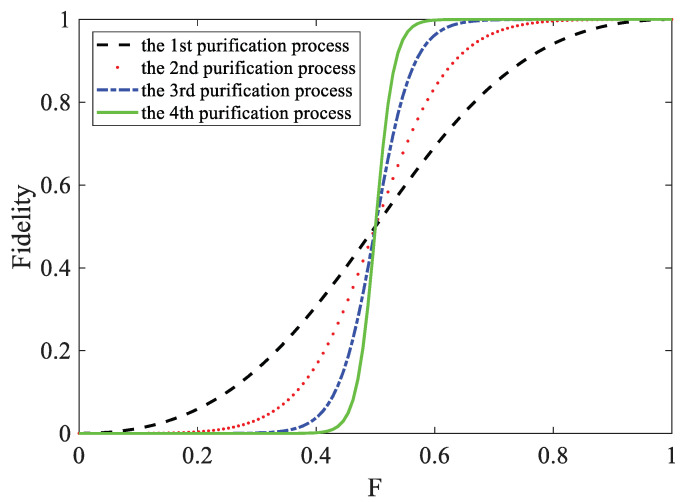
(Color online) Schematic of the fidelity F${}^{\prime }$ of the logic-bell state analysis altered with the initial fidelity F. The blue line represents the first entanglement purification process, the red line represents the second process, the yellow line represents the third process, and the green line represents the fourth process.

We can obtain higher fidelity after more iterations of the purification procedure in the same initial fidelity *F*. The limit of $F^{\left(n\right)}$ is 1 as t→∞, which means an infinite number of cycles of purification. However, in practical applications, we can only perform a finite number of iterations. Fortunately, with the increase of purification times, the fidelity quickly approaches 1. Figure 4 shows the fidelity after one to four steps of purification with *F* from 0 to 1. When the initial fidelity is 0.61, after four cycles of purification, the fidelity can reach 0.999. Users can choose the appropriate number of iterations according to the actual situation.

## 5. Summary

In summary, we have proposed EPPs for four photons’ C-GHZ states with logic bit-flip error and phase-flip error based on PCM gates. The PCM gate in our protocol is realized with a nonlinear cross-Kerr medium and X quadrature measurement, which can distinguish 0 and ±θ with a tiny phase shift and small error probability. In our scheme, the fidelity after each purification is consistent with the optimal result of the same kind of purification scheme [48]. However, since all initial states in $|\Phi^{+}\rangle_{A1B1}⊗{|\Phi^{+}\rangle }_{A2B2}⊗$ and $|\Psi^{+}\rangle_{A1B1}⊗{|\Psi^{+}\rangle }_{A2B2}$ ($|\Phi^{-}\rangle_{A1B1}⊗{|\Phi^{-}\rangle }_{A2B2}$) can be purified, our scheme has a higher purification efficiency. Moreover, the EPPs can be reused for further purification, which can greatly improve the fidelity of the non-local entanglement photon system. These advantages make our EPP more useful for long-distance quantum communication and quantum networks.

## Figures and Tables

**Table 1:** The PCM results for each pair of entanglement states.

Initial States	PCMs Results			
$|\Phi^{+}\rangle_{A1B1}⊗{|\Phi^{+}\rangle }_{A2B2}$,	0000	0011	0101	0110
$|\Psi^{+}\rangle_{A1B1}⊗{|\Psi^{+}\rangle }_{A2B2}$	1111	1100	1010	1001
$|\Phi^{+}\rangle_{A1B1}⊗{|\Psi^{+}\rangle }_{A2B2}$,	0001	0010	0100	0111
$|\Psi^{+}\rangle_{A1B1}⊗{|\Phi^{+}\rangle }_{A2B2}$,	1110	1101	1011	1000

**Table 2:** The operations performed in the output spatial modes $\left\{a_{1}^{\prime },a_{2}^{\prime },b_{1}^{\prime },b_{2}^{\prime }\right\}$ correspond to the measurement results of SPDs in the spatial out modes $\left\{a_{3}^{\prime },a_{4}^{\prime },b_{3}^{\prime },b_{4}^{\prime }\right\}$. *I* and $\sigma_{x}$ are Pauli operations.

Measurement Results	Input States	Output States in Spatial	Operations
of SPDs		Modes $\left\{a_{1}^{\prime },a_{2}^{\prime },b_{1}^{\prime },b_{2}^{\prime }\right\}$	
++++ /−−−−	$|\Phi^{+}\rangle |\Phi^{+}\rangle$	$\frac{1}{\sqrt{2}}\left(|\phi^{+}\rangle |\phi^{+}\rangle +|\phi^{-}\rangle |\phi^{-}\rangle \right)$	I⊗I⊗I⊗I
	$|\Psi^{+}\rangle \langle \Psi^{+}|$	$\frac{1}{\sqrt{2}}\left(|\phi^{+}\rangle |\phi^{-}\rangle +|\phi^{-}\rangle |\phi^{+}\rangle \right)$	
++−− /−−++	$|\Phi^{+}\rangle |\Phi^{+}\rangle$	$\frac{1}{\sqrt{2}}\left(|\phi^{+}\rangle |\phi^{+}\rangle -|\phi^{-}\rangle |\phi^{-}\rangle \right)$	$\sigma_{x}⊗\sigma_{x}⊗I⊗I$
	$|\Psi^{+}\rangle |\Psi^{+}\rangle$	$\frac{1}{\sqrt{2}}\left(|\phi^{+}\rangle |\phi^{-}\rangle -|\phi^{-}\rangle |\phi^{+}\rangle \right)$	
+−+− / −+−+	$|\Phi^{+}\rangle |\Phi^{+}\rangle$	$\frac{1}{\sqrt{2}}\left(|\psi^{+}\rangle |\psi^{+}\rangle +|\psi^{-}\rangle |\psi^{-}\rangle \right)$	$I⊗\sigma_{x}⊗I⊗\sigma_{x}$
	$|\Psi^{+}\rangle |\Psi^{+}\rangle$	$\frac{1}{\sqrt{2}}\left(|\psi^{+}\rangle |\psi^{-}\rangle -|\psi^{-}\rangle |\psi^{+}\rangle \right)$	
+−−+ /−++−	$|\Phi^{+}\rangle |\Phi^{+}\rangle$	$\frac{1}{\sqrt{2}}\left(|\psi^{+}\rangle |\psi^{+}\rangle -|\psi^{-}\rangle |\psi^{-}\rangle \right)$	$I⊗\sigma_{x}⊗\sigma_{x}⊗I$
	$|\Psi^{+}\rangle |\Psi^{+}\rangle$	$\frac{1}{\sqrt{2}}\left(|\psi^{+}\rangle |\psi^{-}\rangle +|\psi^{-}\rangle |\psi^{+}\rangle \right)$	
+++− /−−−+	$|\Phi^{+}\rangle |\Phi^{+}\rangle$	$\frac{1}{\sqrt{2}}\left(|\phi^{+}\rangle |\psi^{+}\rangle -|\phi^{-}\rangle |\psi^{-}\rangle \right)$	$I⊗I⊗\sigma_{x}⊗I$
	$|\Psi^{+}\rangle |\Psi^{+}\rangle$	$\frac{1}{\sqrt{2}}\left(|\phi^{+}\rangle |\psi^{-}\rangle -|\phi^{-}\rangle |\psi^{+}\rangle \right)$	
++−+/ −−+−	$|\Phi^{+}\rangle \langle \Phi^{+}|$	$\frac{1}{\sqrt{2}}\left(|\phi^{+}\rangle |\psi^{+}\rangle +|\phi^{-}\rangle |\psi^{-}\rangle \right)$	$I⊗I⊗I⊗\sigma_{x}$
	$|\Psi^{+}\rangle |\Psi^{+}\rangle$	$\frac{1}{\sqrt{2}}\left(|\phi^{+}\rangle |\psi^{-}\rangle +|\phi^{-}\rangle |\psi^{+}\rangle \right)$	
+−++ /−+−−	$|\Phi^{+}\rangle |\Phi^{+}\rangle$	$\frac{1}{\sqrt{2}}\left(|\psi^{+}\rangle |\phi^{+}\rangle -|\psi^{-}\rangle |\phi^{-}\rangle \right)$	$\sigma_{x}⊗I⊗I⊗I$
	$|\Psi^{+}\rangle |\Psi^{+}\rangle$	$\frac{1}{\sqrt{2}}\left(|\psi^{+}\rangle |\phi^{-}\rangle -|\psi^{-}\rangle |\phi^{+}\rangle \right)$	
−+++ / +−−−	$|\Phi^{+}\rangle |\Phi^{+}\rangle$	$\frac{1}{\sqrt{2}}\left(|\psi^{+}\rangle |\phi^{+}\rangle +|\psi^{-}\rangle |\phi^{-}\rangle \right)$	$I⊗\sigma_{x}⊗I⊗I$
	$|\Psi^{+}\rangle |\Psi^{+}\rangle$	$\frac{1}{\sqrt{2}}\left(|\psi^{+}\rangle |\phi^{-}\rangle +|\psi^{-}\rangle |\phi^{+}\rangle \right)$	

**Table 3:** The PCM results for each pair of entanglement states.

Initial States	PCMs Results
$|\Phi^{+}\rangle_{A1B1}⊗{|\Phi^{+}\rangle }_{A2B2}$,	0000 1111
$|\Phi^{-}\rangle_{A1B1}⊗{|\Phi^{-}\rangle }_{A2B2}$	
$|\Phi^{+}\rangle_{A1B1}⊗{|\Phi^{-}\rangle }_{A2B2}$,	0011 1100
$|\Phi^{-}\rangle_{A1B1}⊗{|\Phi^{+}\rangle }_{A2B2}$,	

**Table 4:** The operations performed in the output spatial modes $\left\{a_{1}^{\prime },a_{2}^{\prime },b_{1}^{\prime },b_{2}^{\prime }\right\}$ correspond to the measurement results of SPDs in the spatial out modes $\left\{a_{3}^{\prime },a_{4}^{\prime },b_{3}^{\prime },b_{4}^{\prime }\right\}$. *I* and $\sigma_{z}$ are Pauli operations.

Measurement Results	Input States	Output States in Spatial	Operations
of SPDs		Modes {a1,a2,b1,b2}	
++++ /−−−−/	$|\Phi^{+}\rangle |\Phi^{+}\rangle$	$\frac{1}{\sqrt{2}}\left(|\phi^{+}\rangle |\phi^{+}\rangle +|\phi^{-}\rangle |\phi^{-}\rangle \right)$	I⊗I⊗I⊗I
++−− /−−++/			
+−+− / −+−+/	$|\Phi^{-}\rangle |\Phi^{-}\rangle$	$\frac{1}{\sqrt{2}}\left(|\phi^{+}\rangle |\phi^{+}\rangle -|\phi^{-}\rangle |\phi^{-}\rangle \right)$	
+−−+ /−++−			
+++− /−−−+/	$|\Phi^{+}\rangle |\Phi^{+}\rangle$	$\frac{1}{\sqrt{2}}\left(|\phi^{+}\rangle |\phi^{-}\rangle +|\phi^{-}\rangle |\phi^{+}\rangle \right)$	$I⊗I⊗\sigma_{z}⊗I$
++−+/ −−+−/			
−+++ / +−−−/	$|\Phi^{-}\rangle |\Phi^{-}\rangle$	$\frac{1}{\sqrt{2}}\left(|\phi^{+}\rangle |\phi^{-}\rangle -|\phi^{-}\rangle |\phi^{+}\rangle \right)$	
+−++ /−+−−			

## References

[1] Ekert A., Jozsa R. (1996). Quantum computation and Shor’s factoring algorithm. Rev. Mod. Phys..

[2] Ekert A.K. (1991). Quantum cryptography based on Bell’s theorem. Phys. Rev. Lett..

[3] Bennett C.H., Brassard G., Mermin N.D. (1992). Quantum cryptography without Bell’s theorem. Phys. Rev. Lett..

[4] Long G.L., Liu X.S. (2002). Theoretically efficient high-capacity quantum-key-distribution scheme. Phys. Rev. A.

[5] Deng F.-G., Long G.L., Liu X.-S. (2003). Two-step quantum direct communication protocol using the Einstein-Podolsky-Rosen pair block. Phys. Rev. A.

[6] Briegel H.J., Dür W., Cirac J.I., Zoller P. (1998). Quantum repeaters: The role of imperfect local Operations in quantum communication. Phys. Rev. Lett..

[7] Stacey W., Annabestani R., Ma X., Lütkenhaus N. (2015). Security of quantum key distribution using a simplified trusted relay. Phys. Rev. A.

[8] Bennett C.H., Brassard G., Crépeau C., Jozsa R., Peres A., Wootters W.K. (1993). Teleporting an unknown quantum state via dual classical and Einstein-Podolsky-Rosen channels. Phys. Rev. Lett..

[9] Calderbank A.R., Rains E.M., Shor P.M., Sloane N.J.A. (1998). Quantum error correction via codes over GF(4). IEEE Trans. Inf. Theory.

[10] Walton Z.D., Abouraddy A.F., Sergienko A.V., Saleh B.E.A., Teich M.C. (2003). Decoherence-free subspaces in quantum key distribution. Phys. Rev. Lett..

[11] Boileau J.C., Gottesman D., Laflamme R., Poulin D., Spekkens R.W. (2004). Robust polarization-based quantum key distribution over a collective-noise channel. Phys. Rev. Lett..

[12] Li X.H., Deng F.G., Zhou H.Y. (2008). Efficient quantum key distribution over a collective noise channel. Phys. Rev. A.

[13] Li C.Y., Li Y.S. (2011). Fault-tolerate three-party quantum secret sharing over a collective-noise channel. Chin. Phys. Lett..

[14] Li C.Y., Li Y.S. (2010). Fault-tolerate quantum key distribution over a collective-noise channel. Int. J. Quantum Inf..

[15] Li C.Y., Zhang Z.R., Sun S.H., Jiang M.S., Liang L.M. (2013). Logic-qubit controlled-NOT gate of decoherence-free subspace with nonlinear quantum optics. J. Opt. Soc. Am. B-Opt. Phys..

[16] Bennett C.H., Bernstein H.J., Popescu S., Schumacher B. (1996). Concentrating partial entanglement by local operations. Phys. Rev. A.

[17] Yamamoto T., Koashi M., Imoto N. (2001). Concentration and purification scheme for two partially entangled photon pairs. Phys. Rev. A.

[18] Zhao Z., Yang T., Chen Y.-A., Zhang A.-N., Pan J.-W. (2003). Experimental realization of entanglement concentration and a quantum repeater. Phys. Rev. Lett..

[19] Sheng Y.-B., Deng F.-G., Zhou H.-Y. (2008). Nonlocal entanglement concentration scheme for partially entangled multipartite systems with nonlinear optics. Phys. Rev. A.

[20] Deng F.-G. (2012). Optimal nonlocal multipartite entanglement concentration based on projection measurements. Phys. Rev. A.

[21] Sheng Y.-B., Zhou L., Zhao S.-M., Zheng B.-Y. (2012). Efficient single-photon-assisted entanglement concentration for partially entangled photon pairs. Phys. Rev. A.

[22] Li C.-Y., Shen Y. (2019). Asymmetrical hyperentanglement concentration for entanglement of polarization and orbital angular momentum. Opt. Express.

[23] Bennett C.H., Brassard G., Popescu S., Schumacher B., Smolin J.A., Wootters W.K. (1996). Purification of noisy entanglement and faithful teleportation via noisy channels. Phys. Rev. Lett..

[24] Pan J.-W., Simon C., Brukner Č., Zeilinger A. (2001). Entanglement purification for quantum communication. Nature.

[25] Sheng Y.-B., Deng F.-G., Zhou H.-Y. (2008). Efficient polarization-entanglement purification based on parametric down-conversion sources with cross-Kerr nonlinearity. Phys. Rev. A.

[26] Sheng Y.B., Deng F.G. (2010). Deterministic entanglement purification and complete nonlocal Bell-state analysis with hyperentanglement. Phys. Rev. A.

[27] Sheng Y.-B., Deng F.-G. (2010). One-step deterministic polarization-entanglement purification using spatial entanglement. Phys. Rev. A.

[28] Li X.-H. (2010). Deterministic polarization-entanglement purification using spatial entanglement. Phys. Rev. A.

[29] Sheng Y.-B., Zhou L. (2015). Deterministic entanglement distillation for secure double-server blind quantum computation. Sci. Rep..

[30] Pan J.-W., Gasparoni S., Ursin R., Weihs G., Zeilinger A. (2003). Experimental entanglement purification of arbitrary unknown states. Nature.

[31] Chen L.-K., Yong H.-L., Xu P., Yao X.-C., Xiang T., Li Z.-D., Liu C., Lu H., Liu N.-L., Li L. (2017). Experimental nested purification for a linear optical quantum repeater. Nat. Photonics.

[32] Kalb N., Reiserer A.A., Humphreys P.C., Bakermans J.J.W., Kamerling S.J., Nickerson N.H., Benjamin S.C., Twitchen D.J., Markham M., Hanson R. (2017). Entanglement distillation between solid-state quantum network nodes. Science.

[33] Wang C., Zhang Y., Jin G.S. (2011). Entanglement purification and concentration of electron-spin entangled states using quantum-dot spins in optical microcavities. Phys. Rev. A.

[34] Yang M., Song W., Cao Z.L. (2005). Entanglement purification for arbitrary unknown ionic states via linear optics. Phys. Rev. A.

[35] Reichle R., Leibfried D., Knill E., Britton J., Blakestad R.B., Jost J.D., Langer C., Ozeri R., Seidelin S., Wineland D.J. (2006). Experimental purification of two-atom entanglement. Nature.

[36] Gonta D., van Loock P. (2012). High-fidelity entanglement purification using chains of atoms and optical cavities. Phys. Rev. A.

[37] Fröwis F., Dür W. (2011). Stable Macroscopic Quantum Superpositions. Phys. Rev. Lett..

[38] Fröwis F., Dür W. (2012). Stability of encoded macroscopic quantum superpositions. Phys. Rev. A.

[39] Kesting F., Fröwis F., Dür W. (2013). Effective noise channels for encoded quantum systems. Phys. Rev. A.

[40] Ding D., Yan F.-L., Gao T. (2013). Preparation of km-photon concatenated Greenberger-Horne-Zeilinger states for observing distinctive quantum effects at macroscopic scales. J. Opt. Soc. Am. B.

[41] Lu H., Chen L.-K., Liu C., Xu P., Yao X.-C., Li L., Liu N.-L., Zhao B., Chen Y.-A., Pan J.-W. (2014). Experimental realization of a concatenated Greenberger–Horne–Zeilinger state for macroscopic quantum superpositions. Nat. Photonics.

[42] Sheng Y.B., Zhou L. (2015). Entanglement analysis for macroscopic Schrödinger’s Cat state. Europhys. Lett..

[43] Sheng Y.B., Zhou L. (2015). Two-step complete polarization logic Bell-state analysis. Sci. Rep..

[44] Zhou L., Sheng Y.-B. (2015). Complete logic Bell-state analysis assisted with photonic Faraday rotation. Phys. Rev. A.

[45] Zhou L., Sheng Y.-B. (2016). Feasible logic Bell-state analysis with linear optics. Sci. Rep..

[46] Zhou L., Sheng Y.B. (2016). Purification of logic-qubit entanglement. Sci. Rep..

[47] Zhou L., Sheng Y.-B. (2017). Polarization entanglement purification for concatenated Greenberger–Horne–Zeilinger state. Ann. Phys..

[48] Wu X.-D., Zhou L., Zhong W., Sheng Y.-B. (2018). Purification of the concatenated Greenberger–Horne–Zeilinger state with linear optics. Quantum Inf. Process..

[49] Pan J., Zhou L., Gu S.-P., Wang X.-F., Sheng Y.-B., Wang Q. (2016). Efficient entanglement concentration for concatenated Greenberger–Horne–Zeilinger state with the cross-Kerr nonlinearity. Quantum Inf. Process..

[50] Nemoto K., Munro W.J. (2004). Nearly deterministic linear optical controlled-NOT gate. Phys. Rev. Lett..

[51] Sheng Y.B., Deng F.G., Zhao B.K., Wang T.J., Zhou H.Y. (2009). Multipartite entanglement purification with quantum nondemolition detectors. Eur. Phys. J. D.

[52] Sheng Y.B., Deng F.G., Zhou H.Y. (2010). Single-photon entanglement concentration for long-distance quantum communication. Quantum Inf. Comput..

[53] Deng F.G. (2011). Efficient multipartite entanglement purification with the entanglement link from a subspace. Phys. Rev. A.

[54] Sun L.L., Wang H.F., Zhang S., Yeon K.H. (2012). Entanglement concentration of partially entangled three-photon W states with weak cross-Kerr nonlinearity. J. Opt. Soc. Am. B.

[55] Liu J., Zhou L., Zhong W., Sheng Y.-B. (2019). Logic Bell state concentration with parity check measurement. Front. Phys..

[56] Li C.Y., Zhang Z.R., Sun S.H., Jiang M.S., Liang L.M. (2013). Optimal symmetric quantum cloning machine with nonlinear optics. J. Opt. Soc. Am. B.

[57] Barrett S.D., Kok P., Nemoto K., Beausoleil R.G., Munro W.J., Spiller T.P. (2005). Symmetry analyzer for nondestructive Bell-state detection using weak nonlinearities. Phys. Rev. A.

[58] Semi<i>a</i>˜o F.L., Vidiella-Barranco A. (2005). Effective cross-Kerr nonlinearity and robust phase gates with trapped ions. Phys. Rev. A.

[59] Hoi I.C., Kockum A.F., Palomaki T., Stace T.M., Fan B.X., Tornberg L., Sathyamoorthy S.R., Johansson G., Delsing P., Wilson C.M. (2013). Giant Cross-Kerr Effect for Propagating Microwaves Induced by an Artificial Atom. Phys. Rev. Lett..

[60] Blais A., Grimsmo A.L., Girvin S.M., Wallraffe A. (2021). Circuit quantum electrodynamics. Rev. Mod. Phys..

